# Galactosidase Alpha p.A143T Variant Fabry Disease May Result in a Phenotype With Multifocal Microvascular Cerebral Involvement at a Young Age

**DOI:** 10.3389/fneur.2018.00336

**Published:** 2018-05-16

**Authors:** Lothar Hauth, Jeroen Kerstens, Laetitia Yperzeele, François Eyskens, Paul M. Parizel, Barbara Willekens

**Affiliations:** ^1^Department of Neurology, Antwerp University Hospital, Antwerp, Belgium; ^2^Faculty of Medicine and Health Sciences, University of Antwerp, Antwerp, Belgium; ^3^Department of Pediatrics, Antwerp University Hospital, Antwerp, Belgium; ^4^Department of Radiology, Antwerp University Hospital & University of Antwerp, Antwerp, Belgium; ^5^Laboratory of Experimental Hematology, Faculty of Medicine and Health Sciences, University of Antwerp, Antwerp, Belgium

**Keywords:** Fabry disease, magnetic resonance imaging, white matter lesions, lysosomal storage disorder, genetic variant of unknown significance

## Abstract

**Introduction:**

A 16-year-old male presented with episodic headaches and a brain magnetic resonance imaging (MRI) that showed multifocal punctate to patchy white matter lesions. The diagnosis of Fabry disease (FD) was suggested upon the finding of significantly reduced plasma alpha-galactosidase A activity (0.62 µmol/L or 13% of normal; normal range ≥ 1.65 μmol/L) and genetic investigation confirmed the presence of a hemizygous missense variant in the galactosidase alpha (GLA) gene (p.A143T). Baseline assessment of other systemic involvement showed only a discrete proteinuria.

**Background:**

FD is a rare lysosomal storage disorder. Genetic screening studies have revealed over 600 variants in the GLA gene. The p.A143T variant is a genetic variant of unknown significance, with its associated phenotype ranging from classical FD to healthy unaffected patients. Some authors, however, deem this variant non-pathogenic. We describe the case of a 16-year-old male with multifocal white matter lesions on brain MRI, who was diagnosed with FD and carried this genetic variant.

**Discussion:**

The causative p.A143T mutation can be associated with a more severe subclinical phenotype than has been reported to date. Furthermore, a diagnosis of FD should be considered when finding asymptomatic cerebral white matter lesions in a young patient.

## Introduction

A 16-year-old Caucasian male was referred to our hospital for further evaluation after the discovery of multiple white matter lesions on brain magnetic resonance imaging (MRI) performed in the diagnostic workup for episodic headaches. Since 1 year, he experienced recurrent, throbbing, biparietal headaches almost exclusively provoked by heat, dehydration or exercise. The headaches were accompanied by general lack of concentration but no other symptoms.

Past medical history included surgical repair of inguinal herniation and varicocoele. He further reported a period at the age of 10 during which he experienced recurrent short episodes of staring and interruption of ongoing activities. Head computed tomography and electroencephalogram at the time showed no significant abnormalities, and these episodes resolved spontaneously after 2 months. He had no history of perinatal asphyxia, serious infections, or cerebral traumata. Family history was unremarkable except for cardiovascular disease on the paternal side. There was no personal or familial history of hypertension. He did not use analgesic medication and was only taking doxycycline since 1 year because of acne. He reported sporadic alcohol use but no prior use of tobacco or other drugs.

**Figure 1:**
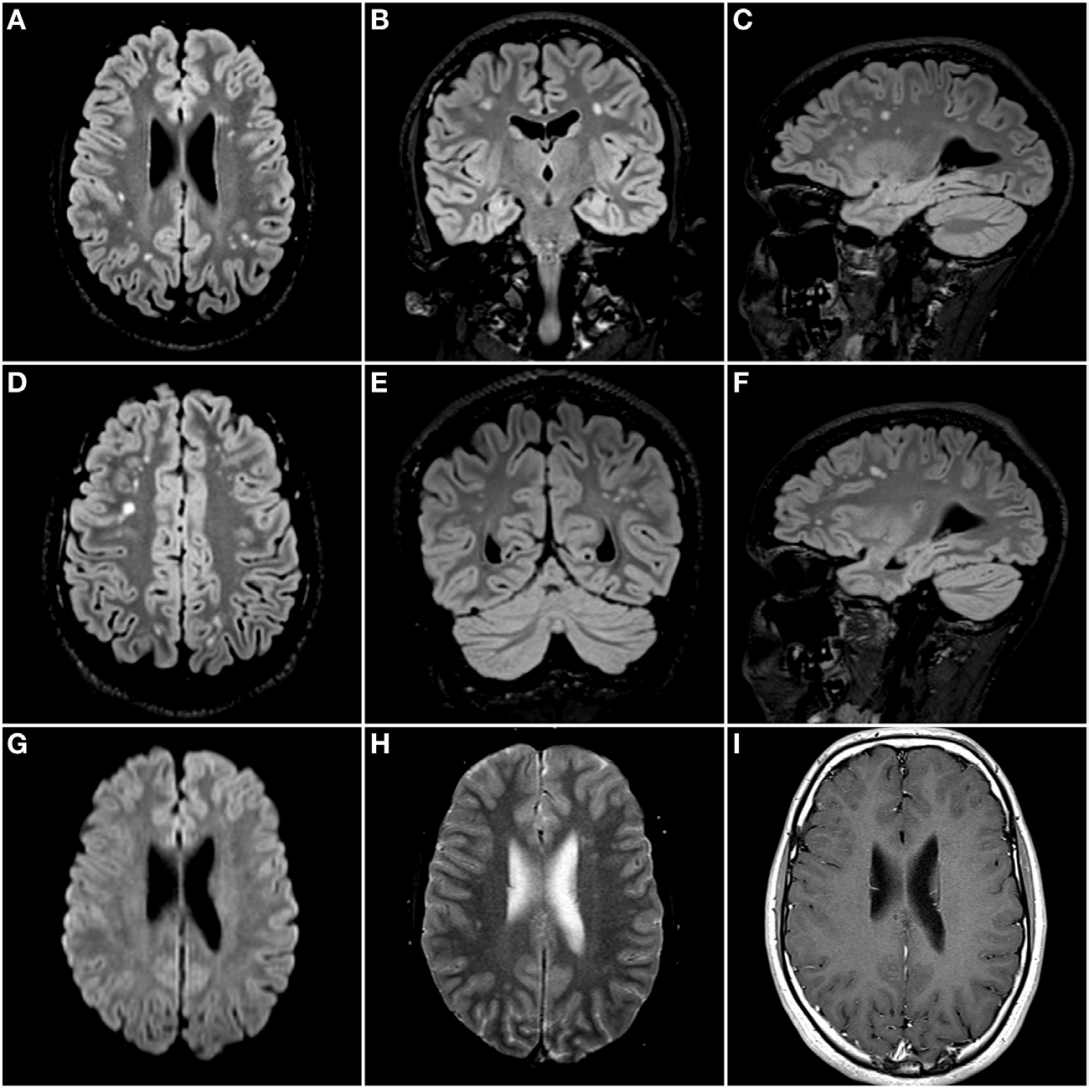
Isotropic 3D Fluid Attenuated Inversion Recovery sequence (TR: 500 ms; TE: 335 ms; and TI: 1,800 ms) with axial **(A,D)**, coronal **(B,E)**, and sagittal **(C,F)** reconstructed images. There are multifocal nodular hyperintense lesions scattered throughout the deep and subcortical white matter of both cerebral hemispheres. There were no areas of diffusion restriction on the diffusion-weighted images **(G)**, nor was there any evidence of microbleeds on the gradient echo T2* images **(H)**. Finally, there was no enhancement after administration of a gadolinium-based contrast agent **(I)**.

Clinical examination showed no abnormalities. Repeat brain MRI scan confirmed the presence of multifocal punctate to patchy white matter lesions (Figure 1), mostly in a subcortical distribution. No significant diffusion restriction, contrast enhancement, microbleeds, or vascular abnormalities were reported.

Extensive blood and CSF examinations did not show evidence for infectious or inflammatory disease. Specifically, antiphospholipid antibodies and genetic testing for CADASIL were negative, and there was no intrathecal synthesis of oligoclonal bands. A chest radiograph was normal. In the laboratory vascular workup, alpha-galactosidase A (AGAL-A) activity was found to be significantly reduced in plasma (0.62 µmol/L or 13% of normal; normal range ≥ 1.65 μmol/L).

The diagnosis of Fabry disease (FD) was confirmed by molecular genetic analysis, showing a hemizygous missense variant in the galactosidase alpha (GLA) gene (p.A143T). Baseline assessment of systemic involvement showed discrete proteinuria (255 mg/24 h; <149) and hyperfiltration (GFR 159.5 mL/min/1.73 m^2^; 90–120) but no other renal, cardiac, ocular, auditory, or dermatological signs’ characteristic for FD. Specifically, a transthoracic echocardiogram and a chest MRI showed no structural abnormalities of the heart. He was subsequently started on enzyme replacement therapy (agalsidase alfa, 0.2 mg/kg intravenously biweekly), which was switched to chaperone therapy (migalastat, 123 mg orally every other day) after approximately 1 year.

His mother was identified as an asymptomatic carrier. His only brother showed the same genetic mutation and a severely decreased AGAL-A activity (<0.05 μmol/L), despite being asymptomatic at age 20. Brain MRI performed 2 years before did not show significant white matter lesions and was not repeated. Further systemic screening was normal apart from a complete right bundle branch block.

## Background

Fabry disease (OMIM301500) is a rare X-linked recessive lysosomal storage disorder with an estimated prevalence of 1 in 17,000 to 1 in 117,000 males (1). It is an inborn error of glycosphingolipid catabolism that results from the deficient activity of the lysosomal enzyme AGAL-A. This leads to accumulation of a-galactosyl-terminal lipids, particularly globotriaosylceramide (Gb3) in lysosomes of different cell types. Both the underlying genetic defect and the clinical presentation of FD are very heterogeneous. Genetic screening studies have revealed more than 600 variants in the GLA gene, most of which appear in single families (2). Such mutations can lead to classical or non-classical forms of FD, and some genetic variants are found to be non-pathogenic or of unknown significance. In classical FD, hemizygous male patients exhibit multiple characteristic features such as acroparesthesia, anhidrosis, disseminated angiokeratoma, cornea verticillata, and (micro)albuminuria. Non-classical FD patients present with late complications, usually restricted to a single organ, such as progressive renal failure, hypertrophic cardiomyopathy, or cerebrovascular disease (3). In this report, we describe a 16-year-old male patient with multiple microvascular cerebral white matter lesions who was found to have the p.A143T variant of the GLA gene.

## Discussion

Clinical manifestations of cerebrovascular involvement in FD include ischemic stroke, transient ischemic attack, intracerebral hemorrhage, subarachnoid hemorrhage, microbleeds, cerebral venous thrombosis, and cervical carotid dissection (4). An analysis of a large cohort of 2,446 patients in the Fabry Registry reported that stroke occurs in 6.9% of male and 4.3% of female patients, with median ages at first stroke of 39.0 and 45.7 years, respectively (5). As in the general population, the incidence of stroke in FD increases with age, with the majority of patients experiencing a first stroke between the age of 20 and 50 years. In the Fabry Registry cohort, 30 of 138 stroke patients (21.7%) had their first stroke at <30 years, including 2 patients who had strokes during their teen years (a 13.8-year-old male and a 19.8-year-old female) (5). In 366 patients in the Fabry Outcome Survey, the mean age at onset of cerebrovascular events was 28.8 years in males and 43.4 years in females, and the youngest patient to report a TIA was a boy aged 12 years (6).

In addition to the aforementioned clinical manifestations of cerebrovascular involvement in FD, many patients have evidence of clinically asymptomatic brain lesions on MRI, most commonly hyperintensities on T2-weighted images of white matter (4). These so-called chronic white matter hyperintensities occur in the subcortical, deep, and periventricular white matter. Several studies have found that 37.5–70.3% of patients with FD present MRI lesions, with a progressive increase in prevalence and lesion load with age (7). To date, only one study evaluated the presence of ischemic and hemorrhagic brain MRI lesions in FD patients without history of stroke or TIA (7). In this series of 46 consecutive patients, 44.4% had subclinical evidence of small vessel disease on brain MRI, but all 10 patients under 20 years of age initially had a normal MRI. One 12-year-old child developed a single hyperintense T2 lesion in the left occipital region at control MRI 1 year later (7). Therefore, our case is remarkable because it describes a 16-year-old patient with multiple hyperintense T2 lesions in both hemispheres. To the best of our knowledge, only two similar cases have been reported before in the medical literature. Cabrera-Salazar et al. described two pediatric male patients with FD discovered upon familial screening (a symptomatic 11-year old and a symptom-free 8-year old) showing multiple punctate areas with increased T2 signal in the subcortical white matter of both hemispheres on MRI, in the absence of clinical evidence of cerebral, renal, or cardiac involvement (8).

Over 600 different mutations are known in the GLA gene coding for AGAL-A. The p.A143T variant present in our patient is considered a genetic variant of unknown significance with its associated phenotype ranging from classical FD to healthy unaffected patients with normal AGAL-A-activity (Table 1). For this reason, its pathogenicity is debated. This missense variant was found in 2 patients in the Belgian Fabry stroke study (BEFAS, 1,000 stroke patients) (9) and in 4 patients in the Stroke in Young Fabry Patients study (SIFAP, 5,023 stroke patients) (10). Furthermore, FD patients in the BEFAS and SIFAP cohorts had no signs of organ involvement other than stroke, TIAs, and headache (10). Therefore, the authors of the latter study hypothesized that the p.A143T variant might be associated with a stroke-only phenotype of FD. De Brabander et al. reported 10 p.A143T carriers (8 females, 2 males). In three females, left ventricular hypertrophy (LVH) was found, without other classical FD signs. They concluded that p.A143T is associated with atypical late-onset FD with predominant cardiac symptoms (11). Yet other authors like Lenders et al. argue that the association between stroke and LVH and the p.A143T variant may be purely coincidental and regard this mutation as non-pathogenic (2, 3, 12, 13). They point out that, in contrast to patients with classical FD, individuals with the p.A143T variant often have considerable residual AGAL-A activity, have no increase in plasma Gb3 and lysoGb3 and biopsies of the affected organs do not find Gb3 storage (2). Unfortunately, neither plasma or urinary Gb3 nor lysoGb3 was assessed in our patient before initiation of enzyme replacement therapy. While one might argue that the normal brain MRI in the brother of our patient is an argument against FD being the cause of the extensive white matter lesions found in this case, this does not exclude a causative relation. Moreover, the p.A143T variant can be associated with different phenotypic expressions (Table 1). Hence, we advocate an individualized approach to patients with the p.A143T.

**Table 1:** Spectrum of systemic involvement in patients with p.A143T galactosidase alpha mutations.

Reference	Neurological involvement	Cardiac involvement	Renal involvement	Other Fabry disease symptoms
(9)	+	−	−	−
(10)	+	−	−	−
(11)	−	+	−	−
(13)	+	−	−	+ (GI symptoms)
(17)	−	+	+	+ (Finger deformities)
(18)	−	−	+	+ (Acroparesthesia)

First, the p.A143T mutation significantly reduces the AGAL-A enzyme activity and although patients with this mutation have a residual AGAL-A activity that is relatively high among all male FD patients, other mutations that have been shown to cause the classical FD phenotype without controversy showed similar residual AGAL-A activity. For example, the missense mutations p.T41I and p.P205T have been found in classically affected male patients (14, 15) and in one study showed a residual AGAL-A activities of 53–61 and 37%, respectively (16). Second, there are several published cases where this mutation is unambiguously disease-causing (17, 18). Altogether, there is no simple answer to the question whether the p.A143T genotype as such is pathogenic or not, because the clinical presentation seems to be highly variable and specific to each case. The likely explanation is that p.A143T indeed has the potential to cause FD, but the relatively high residual AGAL-A activity of this mutant form may also render it subject to the regulatory effect of other genetic modifiers. Therefore, patients with the p.A143T mutation need to be evaluated individually to determine symptomatology and appropriate treatments.

## Concluding Remarks

Summarizing, in our case, the residual AGAL-A activity was low, and the patient had multifocal punctate to patchy white matter lesions. Thus, in our opinion, the GLA p.A143T variant can be associated with a more severe disease type with important subclinical involvement of the brain than has been previously reported. In case of incidental multifocal white matter lesions with a vascular distribution in young patients, FD should be considered in the differential diagnosis, even when there is no family history.

## Informed Consent

Written informed consent was obtained from the participant for the publication of this case report.

## Ethics Statement

No approval from an ethics committee was obtained: the paper presented is a case report with a study of available literature. All subjects gave written informed consent in accordance with the Declaration of Helsinki.

## Author Contributions

All the authors contributed to the conception and design or analysis and interpretation of data. LH and JK drafted the article and LY, PP, FE, and BW revised it critically for important intellectual content.

## Conflict of Interest Statement

LH: has received travel grants and registration fees by Shire. JK, PP, and BW: no competing interests declared. LY: has received registration fees and study funding by Shire. FE: has received consulting and speaking fees by Sanofi-Genzyme and registration fees by Amicus.
